# Differential Effects of IGF-1R Small Molecule Tyrosine Kinase Inhibitors BMS-754807 and OSI-906 on Human Cancer Cell Lines

**DOI:** 10.3390/cancers12123717

**Published:** 2020-12-11

**Authors:** María Fuentes-Baile, María P. Ventero, José A. Encinar, Pilar García-Morales, María Poveda-Deltell, Elizabeth Pérez-Valenciano, Víctor M. Barberá, Javier Gallego-Plazas, Álvaro Rodríguez-Lescure, José Martín-Nieto, Miguel Saceda

**Affiliations:** 1 Unidad de Investigación, Fundación para el Fomento de la Investigación Sanitaria y Biomédica de la Comunidad Valenciana (FISABIO), Hospital General Universitario de Elche, 03203 Elche (Alicante), Spain; fuentes_marbai@gva.es (M.F.-B.); barbera_vicjua@gva.es (V.M.B.); 2 Unidad de Investigación, Instituto de Investigación Sanitaria y Biomédica de Alicante (ISABIAL), Hospital General Universitario de Alicante, 03005 Alicante, Spain; ventero_marmar@gva.es; 3 Instituto de Biología Molecular y Celular (IBMC) and Instituto de Investigación, Desarrollo e Innovación en Biotecnología Sanitaria de Elche (IDiBE), Universidad Miguel Hernández, 03202 Elche (Alicante), Spain; pgarcia@umh.es (P.G.-M.); mpoveda@inveready.com (M.P.-D.); elizabeth.perez@goumh.umh.es (E.P.-V.); 4 Unidad de Genética Molecular, Hospital General Universitario de Elche, 03203 Elche (Alicante), Spain; 5 Servicio de Oncología, Hospital General Universitario de Elche, 03203 Elche (Alicante), Spain; jgallego@umh.es (J.G.-P.); arodriguez@umh.es (Á.R.-L.); 6 Departamento de Fisiología, Genética y Microbiología, Facultad de Ciencias, Universidad de Alicante, 03080 Alicante, Spain; jmnieto@ua.es

**Keywords:** IGF-1R inhibitor, ATP-binding domain, off-target inhibition, molecular docking, pancreatic carcinoma, colon carcinoma, glioblastoma, tyrosine kinase

## Abstract

**Simple Summary:**

We have tested the effects of IGF-1R tyrosine kinase inhibitors BMS-754807 (BMS) and OSI-906 (OSI) on human colon, pancreatic carcinoma cell, and glioblastoma cell lines and primary cultures. Although OSI and BMS are able to inhibit IGF-1R activity at low doses, the differential effect on cell proliferation and cell-cycle phase distribution shown by both compounds probes that many effects observed are mediated by BMS off-target interactions. Using MAPKs ELISAs and phospho-RTK array analysis, we have identified several BMS regulated putative kinases able to mediate BMS off-target effects. Interestingly, molecular docking assays suggest that BMS could affect these kinases not only by blocking their ATP-binding domain, but also by means of allosteric interactions. Since BMS has an important antineoplastic effect on these poor prognosis types of cancer, these compounds could be taken in consideration for treatment independently of IGF-1R status.

**Abstract:**

We have determined the effects of the IGF-1R tyrosine kinase inhibitors BMS-754807 (BMS) and OSI-906 (OSI) on cell proliferation and cell-cycle phase distribution in human colon, pancreatic carcinoma, and glioblastoma cell lines and primary cultures. IGF-1R signaling was blocked by BMS and OSI at equivalent doses, although both inhibitors exhibited differential antiproliferative effects. In all pancreatic carcinoma cell lines tested, BMS exerted a strong antiproliferative effect, whereas OSI had a minimal effect. Similar results were obtained on glioblastoma primary cultures, where HGUE-GB-15, -16 and -17 displayed resistance to OSI effects, whereas they were inhibited in their proliferation by BMS. Differential effects of BMS and OSI were also observed in colon carcinoma cell lines. Both inhibitors also showed different effects on cell cycle phase distribution, BMS induced G_2_/M arrest followed by cell death, while OSI induced G_1_ arrest with no cell death. Both inhibitors also showed different effects on other protein kinases activities. Taken together, our results are indicative that BMS mainly acts through off-target effects exerted on other protein kinases. Given that BMS exhibits a potent antiproliferative effect, we believe that this compound could be useful for the treatment of different types of tumors independently of their IGF-1R activation status.

## 1. Introduction

IGF-1R (UniProtKB code P08069) is a tyrosine kinase receptor located in the plasma membrane, which is involved in the processes of cell growth, development, and differentiation. In addition, it exhibits a very strong antiapoptotic activity [1]. The *IGF1R* gene is translated into a single polypeptide precursor that is cleaved to yield an α subunit, which contains the ligand-interacting domain, and a β subunit, which contains the transmembrane and tyrosine kinase domains [2]. These two subunits remain linked by disulfide bonds, the structure of the receptor being a heterotetramer with a βααβ conformation [3]. The receptor can also form hybrid heterotetramers with the α and β chains of the insulin receptor (IR) [2]. Its ligands are insulin-like growth factors 1 and 2 (IGF-1 and IGF-2) and insulin. IGFR-1R binds IGF-1 with high affinity, and IGF-2 and insulin with lower affinity [4]. Activation of IGF-1R upon ligand binding leads to the autophosphorylation of its tyrosine kinase domain, with ensuing activation of the Ras-Raf-MAPK and PI3K-AKT/PKB signaling pathways, which is crucial for IGF-1R to exert its mitogenic and antiapoptotic activities [3].

Numerous studies have shown that IGF-1R is overexpressed in primary tumors and cancer-derived cells. This increase in IGF-1R levels reflects a reversion to more primitive, less differentiated and oncogenic states that are characterized by high concentrations of IGF-1R mRNA and IGF-binding sites [4]. In this context, it has been shown that active IGF-1R can be found overexpressed in all subtypes of breast cancer, and that the presence of high levels of phosphorylated IGF-1R is associated with a lower patient survival [5]. Overexpression of IGF-1R has also been associated with a higher tumor grade, inhibition of apoptosis, increased proliferation rate, and angiogenesis in patients with pancreatic ductal adenocarcinoma [6], and with a lower survival in patients with colorectal cancer [7]. Moreover, the IGF-1R signaling pathway is highly active in different types of human tumors, as is the case for metastatic melanoma [8], and is known to play a critical role in the transformation, growth and survival of glioblastoma multiforme (GBM) cells [9,10].

Currently, strategies are being developed in order to exploit IGF-1R as a therapeutic target [3]. Inhibitors of IGF or IGF-1R are being tested in clinical trials that belong to three main classes: monoclonal antibodies against IGF-1R, monoclonal antibodies against IGF-1R ligands (IGF-1 and IGF-2), and IGF-1R tyrosine kinase inhibitors [11]. Agents that target IGF-1R include monoclonal antibodies such as cixutumumab (IMC-A12), dalotuzumab (MK-0646) and robatumumab (Sch717454), and the small molecules acting as tyrosine kinase inhibitors dubbed BMS-754807 (BMS-754807), linsitinib (OSI-906), XL228 and AXL1717 [12]. Among these, BMS and OSI-906 (OSI) are taken orally and constitute the most specific, ATP-competitive inhibitors, whereas others also inhibit receptor tyrosine kinases beyond the IGF-1R and IR family [11]. BMS-754807 is a potent and reversible inhibitor of both IGF-1R and IR, with a half-maximal inhibitory concentration (IC50) of 1.8 nM and 1.7 nM, respectively, in cell-free assays [13]. On the other hand, it is less potent on Met, Aurora A/B, TrkA/B and Ron, and shows little activity on Flt3, Lck, MK2, PKA, PKC and other protein kinases [14]. BMS-754807 effectively inhibits the growth of a wide range of human tumor types in vitro, including mesenchymal (Ewing sarcoma, rhabdomyosarcoma, neuroblastoma, and liposarcoma), epithelial (breast, lung, pancreas, colon, gastric), and hematopoietic (multiple myeloma and leukemia) tumor cell lines. It has been shown that this compound causes apoptosis in a human rhabdomyosarcoma cell line, associated with an increased cleavage of poly ADP-ribose polymerase (PARP) and caspase-3 expression [13]. Regarding OSI-906, this compound is a selective inhibitor of IGF-1R, with an IC50 of 35 nM in cell-free assays, and is modestly potent against the IR, with an IC50 of 75 nM. It is also known to have no activity towards Abl, ALK, BTK, EGFR, FGFR1/2, PKA and other protein kinases [15,16]. OSI-906 inhibits the proliferation of hepatocellular carcinoma (HCC) cell lines by at least 40%. HCC cells sensitive to OSI-906 show higher levels of phosphorylation of IGF-1R and IR than resistant cells, suggesting that sensitivity to OSI-906 is associated with the inhibition of both of these receptors [17]. Moreover, OSI-906-induced apoptosis and inhibition of cell proliferation appear to be directly linked to the inhibition of AKT in several tumor cell lines, including lung, pancreas and colorectal cell lines [18]. In this context, OSI-906-treated colorectal cancer xenografts show a decrease in tumor growth and increased apoptosis in vivo and in vitro [19]. In this system, OSI-906 has been found to ameliorate cell proliferation by altering the cell cycle in the G_0_/G_1_ phase.

In this work, we have addressed the effects of BMS-754807 and OSI-906 on cell proliferation and cell-cycle phase distribution in several human colon, and pancreatic carcinoma, and glioblastoma cell lines and primary cultures derived from glioblastoma patients. Our results show that BMS-754807 mainly acts through off-target effects exerted on other protein kinases independently of IGF-1R inhibition. Given that BMS-754807 exhibits a potent antiproliferative effects on glioblastoma, colon and pancreatic carcinoma cellular models analyzed in this work, we believe that this compound could be useful for the treatment of different types of tumors independently of their IGF-1R activation status.

## 2. Results

### 2.1. BMS-754807 and OSI-906 Effect on IGF-1R Phosphorylation

**Figure 1:**
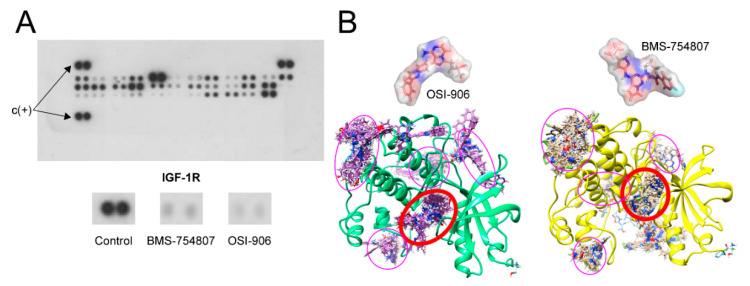
(**A**) Representative image of a human phospho-RTK array (R&D Systems) analysis performed on RWP-1 cells. The three panels below show a magnification of the IGF-1R dots obtained from RWP-1 cells grown in the presence of 10% FBS, control (untreated) and treated for 3 h with 10 µM OSI-906 or BMS-754807, C(+) show the positive control spots used for the normalization of fluorescence intensity between different filters. (**B**) Docking analysis of the interaction between the two inhibitors, BMS-754807 and OSI-906, on the IGF-1R structure. Purples ellipses represent alternative theoretical binding sites to IGF-1R and red ellipse represent the ATP-binding domain, which is also the highest affinity site in both cases.

Given that both compounds, BMS and OSI, have been developed as IGF-1R and IR inhibitors, we decided to study whether equivalent doses of these drugs were able to inhibit IGF-1R phosphorylation to a similar extent. With this purpose, we tested the effects of a 10 µM dose of BMS or OSI on IGF-1R phosphorylation in the human pancreatic cell lines IMIM-PC-2 and RWP-1. As shown in Figure 1A, both inhibitors were able to block almost completely IGF-1R phosphorylation induced by 10% FBS, as determined by using a commercial human phospho-RTK array. In addition, IGF-1R phosphorylation was analyzed by western blotting using antibodies against phospho-IGF-1R (Tyr-857) or IGF-1R. OSI and BMS were used at 500 nM and 10 µM (Figure S1). Both compounds were able to inhibit IGF-1R phosphorylation at low doses.

Next, we assessed by molecular docking assays the interaction between both inhibitors, BMS and OSI, on the IGF-1R protein structure, as shown in Figure 1B. Both compounds were predicted to bind preferentially to the ATP-binding site of this receptor with equivalent affinities. Moreover, the calculated Gibbs free energy changes (∆G) for both compounds were quite similar, of −9.75 and −9.45 kcal/mol for BMS and OSI, respectively.

### 2.2. BMS-754807 and OSI-906 Effects on Cell Viability

**Figure 2:**
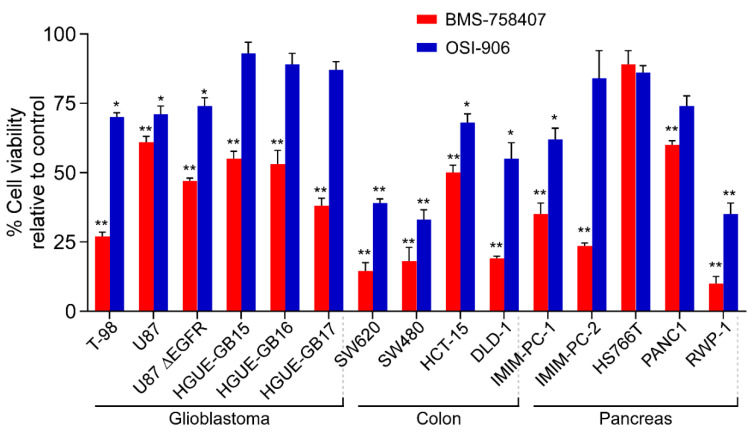
BMS-754807 and OSI-906 effect on cell viability in glioblastoma, colon and pancreatic cancer cell lines. The indicated cell lines were treated with 10 µM BMS-754807 or OSI-906 for 72 h, and cell proliferation was evaluated by MTT assays. Data represent the mean ± SEM (*n* ≥ 6) of viable cell percentage in the presence of 10 µM BMS or OSI compounds, as compared to untreated cells taken as 100%. *, *p* < 0.05; **, *p* < 0.01.

**Figure 3:**
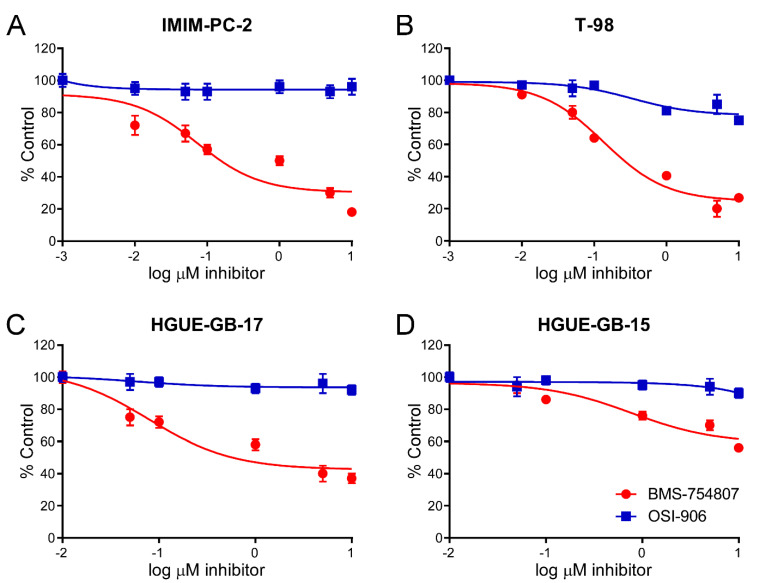
Dose–response effect of BMS-754807 and OSI-906 on cell proliferation in different tumor cell lines. The indicated cell lines were treated with 0.01–10 µM BMS-754807 or OSI-906 for 72 h and cell proliferation was evaluated by the MTT assay. (**A**) IMIM-PC-2 pancreatic carcinoma cell line; (**B**) T98 glioblastoma cell line; (**C**,**D**) HGUE-GB-17 and HGUE-GB-15 glioblastoma primary cultures, respectively. The solid line of each plot has been calculated by fitting the three parameters of a sigmoid equation (dose–response curve) on the data that represent the decimal logarithm of the inhibitor concentration versus the response obtained. GraphPad Prism 5 software (GraphPad Software Inc., San Diego, CA, USA) has been used. Data represent the mean ±SEM (n ≥ 6) of viable cells percentage with respect to untreated controls, taken as 100%.

In order to analyze the effect of both IGF-1R inhibitors on cell lines derived from different types of human tumors, we carried out MTT cell-proliferation assays in the presence of BMS or OSI. The results shown in Figure 2 illustrate that the decrease in the percentage of viable cells after treatment with 10 µM BMS or OSI in different glioblastoma, colon and pancreatic carcinoma cell lines was quite different for the two inhibitors, with the result that BMS had a stronger inhibitory effect on cell growth in almost all cell lines tested as compared with OSI. Indeed, several cell lines were resistant to OSI but were inhibited by BMS, which was especially evident for the three glioblastoma primary cultures and the IMIM-PC-2 pancreatic carcinoma cell line. In general, 10 µM OSI inhibited cell growth by 10–40%, whereas the same concentration of BMS caused a 40–80% inhibition, depending on the cell line. We also performed MTT assays using different concentrations of both inhibitors ranging from 0.1 to 10 µM on all the cellular models studied. The results obtained in dose–response experiments carried out in four cell lines are shown in Figure 3 and in two more cell lines, SW480 and RWP-1 in Figure S2. These results demonstrate that the differential effects of the two compounds on cell proliferation were manifest at all concentrations tested.

### 2.3. BMS-754807 and OSI-906 Effects on Cell Cycle Phase Distribution

**Figure 4:**
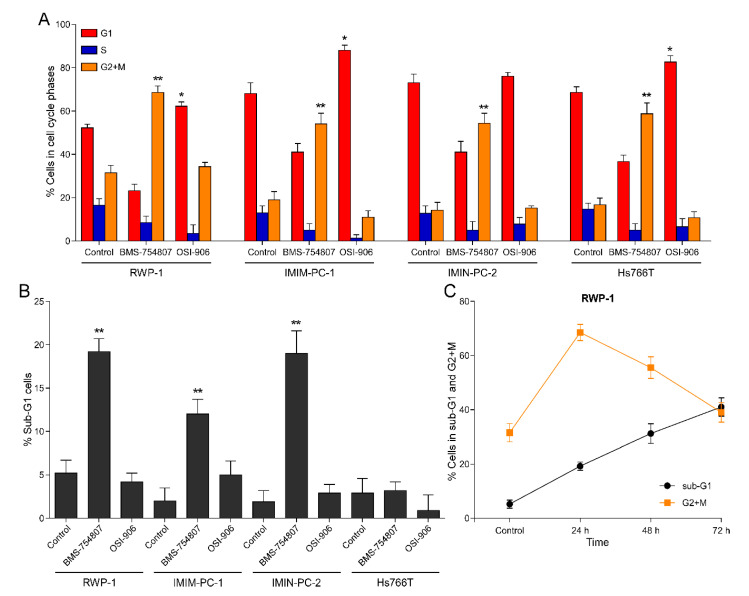
(**A**) Effect of BMS-754807 and OSI-906 on cell cycle phase distribution in pancreatic carcinoma cell lines. RWP-1, IMIM-PC-1, IMIM-PC-2 and HS766T cell lines were treated with 10 µM BMS-754807 or OSI-906 for 24 h and cell cycle phase distribution was analyzed by flow cytometry. Data represent the mean ± SEM (*n* ≥ 3) of the percentage of cells in each phase of the cell cycle. * *p* < 0.05, ** *p* < 0.01. (**B**) Cell death induction by BMS-754807 and OSI-906 in pancreatic carcinoma cell lines. RWP-1, IMIM-PC-1, IMIM-PC-2 and HS766T were treated with 10 BMS-754807 µM or OSI-906 for 24 h. Data represent the mean ± SEM (*n* ≥ 3) of the percentage of dead cells (sub-G1) on the cell cycle analysis represented in A. ** *p* < 0.01. (**C**) Effect of 10 µM BMS-754807 for 24, 48 and 72 h in the pancreatic carcinoma cell line RWP-1. Data represent the increase in the percentage of cell death and the parallel decrease in cells in the G2 + M phase of the cell cycle. Data represent the mean ± SEM (*n* ≥ 3).

To determine the effect of both inhibitors, BMS and OSI, on the distribution of cells among the different phases of the cell cycle, they were treated or not with 10 µM BMS or OSI for 24 h, and then their DNA was labeled with propidium iodide. Figure 4A shows the results obtained by flow cytometry in the four pancreatic carcinoma cell lines analyzed, reflecting that the two inhibitors exerted differential effects on cell cycle phase distribution. While OSI produced no effect or blockade in the G_1_ phase, BMS elicited an arrest in the G_2_+M phases of the cell cycle. BMS also causes an increase (albeit small) in the fraction of cells in sub-G_1_ phase (Figure 4B), which was indicative of cell death. Similar results were obtained in colon and glioblastoma cell models. The sub-G_1_ phase after BMS treatment shown in Figure 4B is statistically significant although small; however, it has to be taken into account that the maximum effect of OSI and BMS is shown in Figure 3, which presents the MTT test data carried out after 72 h of treatment. After 24 h of BMS treatment, a percentage of cells in the sub-G_1_ phase is observed, but also a much higher fraction is blocked in the G2 + M phase, which are already marked to die. If we follow the effect on the cell cycle at 48 and 72 h, cells blocked in G2 + M are translocated to the Sub-G1 phase (Figure 4C).

**Figure 5:**
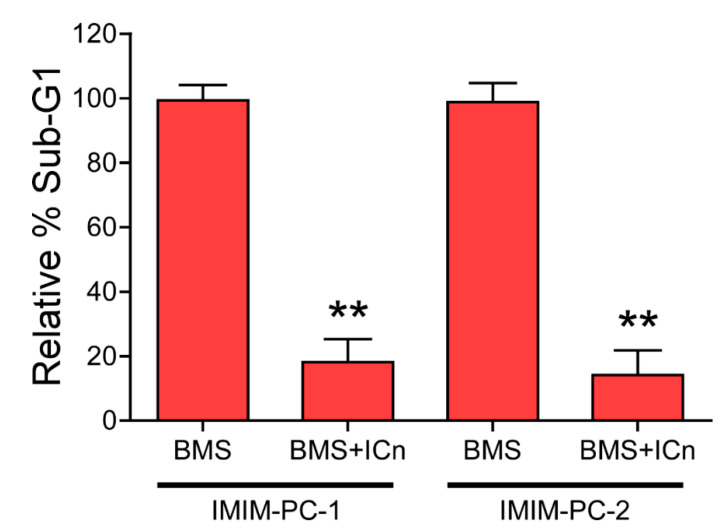
Effect of a pan-caspase inhibitor on cell death induced by BMS-754807. IMIM-PC-1 and IMIM-PC-2 cell lines were treated with 10 µM BMS-754807 (BMS) in the presence or absence of 25 µM Z-VAD-FMK (ICn) for 24 h, and the number of cells in the sub-G_1_ phase of the cell cycle was determined by flow cytometry. Data represent the mean ± SEM (*n* ≥ 3), taking the number of BMS-treated, ICn-untreated cells in sub-G_1_ phase as 100%. **, *p* < 0.01.

We have previously studied the effect of another inhibitor of IGF-1R, picropodophyllin (PPP), on glioblastoma cellular models, and determined that the molecular mechanism of cell death induced by this compound was not a caspase-dependent apoptosis [20]. Accordingly, we decided to test a pan-caspase inhibitor in order to determine whether BMS-induced cell death occurred or not by means of caspase-dependent apoptosis. Figure 5 shows the effect of the general caspase inhibitor, Z-VAD-FMK, on BMS-754807-induced cell death in IMIM-PC-1 and IMIM-PC-2 pancreatic carcinoma cell lines. Our results reflected that this pan-caspase inhibitor almost completely abolished cell death induced by BMS, suggesting that this phenomenon took place through a typical caspase-dependent apoptotic mechanism. This is an interesting finding since we have tested three putative inhibitors of IGF-1R in this and in our previous article, and we have found that two of them (namely PPP and BMS) induced cell death and blocked cell cycle in the G2 + M phase, although their mechanisms of cell death induction were different (one is caspase dependent, while the other one is caspase independent) [20].

### 2.4. BMS-754807 and OSI-906 Effects on the Activity of Intracellular Protein Kinases

**Figure 6:**
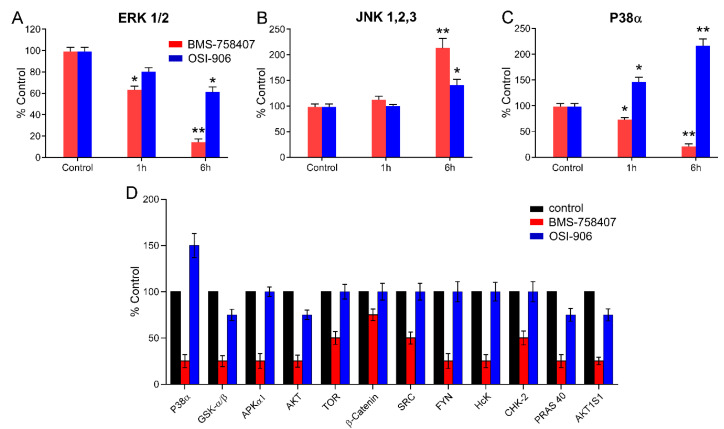
Effect of BMS-754807 and OSI-906 on the activation of off-target protein kinases. The RWP-1 pancreatic carcinoma cell line was treated for 1 or 6 h with 10 µM BMS-754807 or OSI-906. ERK ½ (**A**), JNK 1, 2, 3 (**B**) and p38α (**C**) were determined by using an InstantOne ELISA kit from eBioscience. (**D**). RWP-1 cells were treated with 10 µM BMS-754807 or OSI-906, or left untreated for 6 h, and then subjected to analysis on a human phosphor-RTK array. The graph shows the effect of both inhibitors on the phosphorylation status of the different protein kinases included in the array. Data represent the mean ± SEM (n ≥ 3) of phosphorylation levels, taking those of untreated cells as 100%. *, *p* < 0.05; **, *p* < 0.01.

Given that both compounds, BMS and OSI, are inhibitors of IGF-1R tyrosine kinase activity, we set to assess whether their observed differential effects on cancer cell lines were attributable to their possible ability to inhibit off-target protein kinases. With this purpose, we started by analyzing their putative effect on the activity of different MAP kinases. We performed these experiments in the pancreatic carcinoma cell line RWP-1, since both compounds inhibited their proliferation and affected their cell cycle phase distribution (Figure S2 and Figure 4A,B). An ELISA test was used to determine the phosphorylation (and hence activation) status of a set of MAP kinases, namely, ERK 1 and 2, JNK 1, 2 and 3, and p38α, after 1 and 6 h of treatment with 10 µM BMS or OSI. Since we were looking for putative alternative targets of BMS, independently of IGF-1R, to maximize the differential targets of OSI and BMS we use a high dose of these compounds, 10 µM. It is obvious that when we treat cells in culture, we could add very low doses, but this would not be the real situation concerning the doses of these drugs received by the patients that, obviously, are not going to receive nM doses, so we need to study the effects of real doses, in order to mimic the situation in the tumor cells inside the patients. There are several articles that study the concentration of linsitinib (OSI) in plasma and blood of human patients. The concentrations of linsitinib in the plasma range from 1789 ng/mL (approx. 4.5 µM) to values higher than 4500 ng/mL (approx. 10.6 µM), which means that our data with 10 µM just to maximize the difference between OSI and BMS to allow us identify BMS off targets is in the range of the dose received by the patient [21,22], not to mention the doses used in mouse xenograft models, where OSI and BMS are used in doses as high as 40 mg/kg, that is, a dose equivalent to several hundred µM. Our results shown in Figure 6A reflected that both compounds negatively affected ERK1/2 phosphorylation, although BMS exerted a stronger inhibitory effect (by 80%) than OSI (by 30%). On the other hand, both compounds elicited an increase in the phosphorylation of JNK1-3 (Figure 6B), again BMS being more effective (with a 100% increase over control phosphorylation) than OSI (40% increase). The greatest difference between the two compounds was observed on the activation of p38α (Figure 6C), where OSI induced a higher than 100% increase in its phosphorylation over control levels, whereas BMS resulted in the inhibition of p38α phosphorylation by 80%.

In an attempt to identify possible differential targets for BMS and OSI, we used a human phospho-kinase array. The most significant results of these experiments are shown in Figure 6D, which validated the differential effect of both compounds on p38α, but additionally allowed us to identify a number of protein kinases, such as GSK-3, AMPK, AKT, SRC, CHK2, among others, whose phosphorylation was inhibited by BMS, but not by OSI. These data pointed out that many of the differential effects observed between OSI and BMS on our tested cell lines were due to inhibition promoted by BMS, but not by OSI, of two main intracellular signaling pathways, namely, PI3K/AKT/mTOR and TP53. In addition, Western blot analysis of BMS and OSI effects on ERK ½ and AKT in RWP-1 cells was performed, showing the same results.

### 2.5. BMS-754807 and OSI-906 Potential Interaction with Protein Kinases

**Figure 7:**
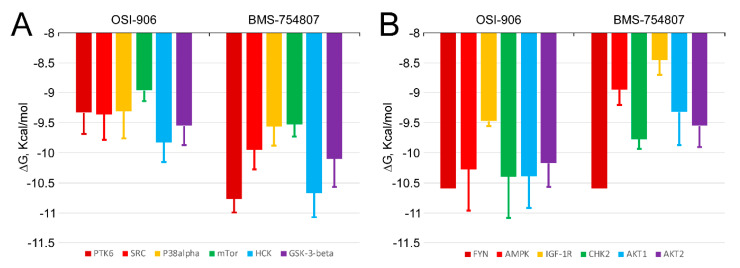
Comparison of Gibbs free energy variation (ΔG, kcal/mol) for BMS-754807 and OSI-906 inhibitors based on molecular docking against the ATP-binding site of several protein kinases (see Table S1 for their UniProtKB accession numbers). The data have been distributed in two panels (**A**,**B**) to facilitate comparison. Panel **A** shows the results of molecular docking analyses for PTK6, SRC, p38α, mTOR, HCK and GSK-3β protein kinases. Panel **B** shows the results for FYN, AMPK, IGF-1R, CHK2, AKT1 and AKT2.protein kinases.

**Figure 8:**
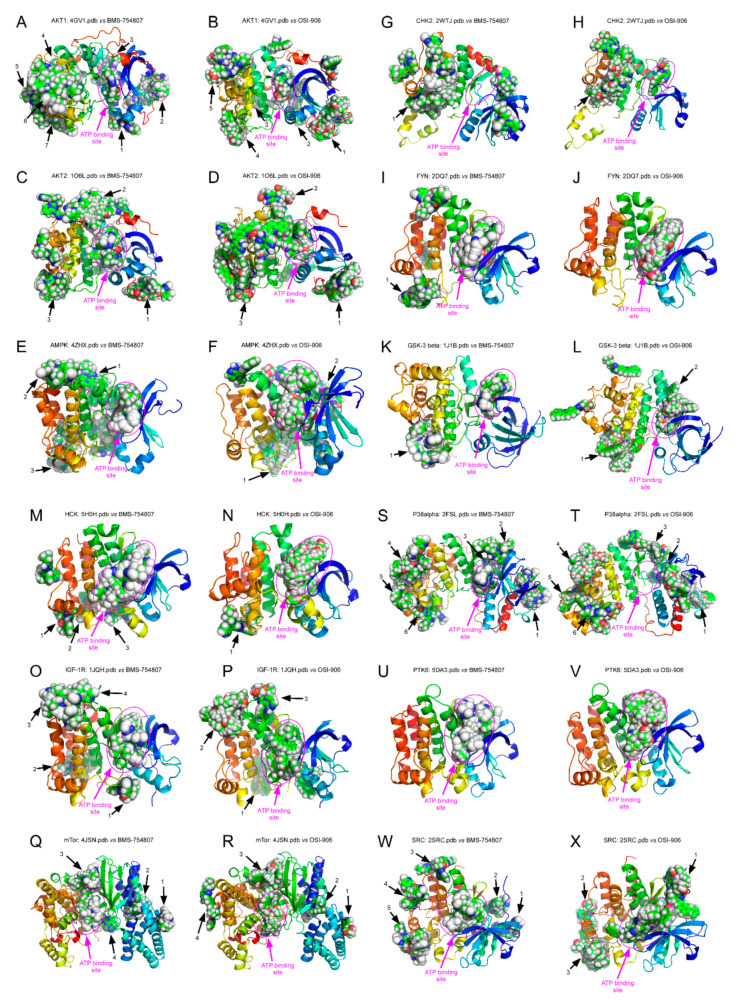
Molecular docking of BMS-754807 and OSI-906 binding to the full catalytic domain of several protein kinases. In each panel, the catalytic domain of each indicated protein kinase was represented by its amino acid backbone as a colored cartoon in the rainbow range from N-terminal (**blue**) to C-terminal (**red**). A pink ellipse delineates the ATP-binding site, and the ligand molecules are represented by Van der Waals spheres. Numbered black arrows for each ligand-protein kinase binding area (cluster), different from the ATP-binding site, are also indicated.

Figure 7 shows the free energy variation (ΔG, kcal/mol) calculated using AutoDock/vina for the best docking scores of BMS-754807 and OSI-906 interaction with the ATP-binding site in the catalytic domain of several protein kinases identified in the phosphor-kinase array assay. The calculated K_D_ (K_D_ = exp^ΔG/RT^) for compounds with a ΔG ≤ −10.5 kcal/mol was in the nanomolar or subnanomolar range [23,24]. As noticeable from values shown in Figure 7, only BMS-754807 displayed a ΔG below that value for its binding to PTK6, HCK (panel A) and FYN (panel B). The ΔG values were between 0.5 and 1.5 kcal/mol greater for OSI-906 than for BMS-754807, which would imply a higher affinity of the latter for the ATP-binding site of protein kinases PTK6, SRC, p38α, mTOR, HCK, GSK-3β (Figure 7A) and IGF-1R (Figure 7B). These data were in agreement with our experimental observations from phosphor-RTK array analysis, showing that whereas BMS-754807 clearly inhibited these enzymes, OSI-906 did not, or did it less effectively (Figure 6D). However, for some of the protein kinases shown in Figure 7B, namely, AMPK, CHK2, AKT1 and AKT2, the ΔG values for OSI-906 binding were lower than for BMS-754807, implying that the affinity of the latter for their ATP-binding site would be higher. In this last case, the molecular docking data were in disagreement with the experimental data from phosphor-kinase arrays. Both drugs have been designed against the ATP-binding site and should thus behave as competitive inhibitors. However, OSI and BMS could bind with high affinity to other areas different from the ATP-binding site, and thereby exert a role as allosteric modulators or by preventing interactions with other proteins acting in upstream or downstream signaling cascades in which the studied protein kinases also participate. In order to address this question, we carried out 500 runs of molecular docking assays for BMS-754807 and OSI-906 interactions with the full catalytic domains of all the 12 protein kinases indicated in Figure 7, with the results depicted in Figure 8. For each protein kinase, we found a different number of clusters of interaction sites, ranging from one to five, which differed by <5 Å in their root mean square deviation values for BMS-754807 and OSI-906 compounds. A first unexpected observation was that the molecular docking assays did not show any hotspot (cluster) for BMS-754807 or OSI-906 interaction with the ATP-binding sites of CHK2 (Figure 8G) or p38α (Figure 8T), respectively. In general, these docking assays revealed a greater number of hotspots for BMS-754807 than for OSI-906, which could explain the more effective inhibitory effect displayed on cells by BMS-754807 in our experimental data. Also noticeable was the presence of clusters for binding of this inhibitor in the area of intersection between the N- and C-terminal domains of AKT2 (Figure 8C), AMPK (Figure 8E), mTOR (Figure 8Q), p38α (Figure 8S) and SRC (Figure 8W). It is tempting to speculate that BMS-754807 binding to this area would prevent the necessary opening and closing movement around the cleft in the catalytic domain necessary for ATP binding.

## 3. Discussion

This work shows that both BMS and OSI small molecules indeed act as inhibitors of IGF-1R, as previously reported [2,11,19,25,26], after performing tests using two immunological techniques, namely protein-RTK arrays and Western blotting, and additionally through molecular modeling analyses (Figure 1). In addition, we demonstrate that, as reported for both BMS-754807 [13,26,27] and OSI-906 [19], in our hands, these two compounds exerted antiproliferative effects on cell lines derived from different types of cancer and on tumor primary cultures (Figure 2 and Figure 3). However, treatment with BMS-754807 in general elicited a more effective antiproliferative effect (by 60–80%) on cancer cells than treatment with OSI-906 (by 30–40%). Moreover, several cell lines revealed to be resistant to OSI-906, but sensitive to BMS-754807, including the IMIM-PC-2 (M220) pancreatic carcinoma cell line and the glioblastoma primary cultures HGUE-GB-15, HGUE-GB-16 and HGUE-GB- 17. These facts lead us to think that BMS-754807, in addition to inhibiting IGF-1R and IR, could bear alternative protein kinase targets which could explain its stronger antiproliferative effect as compared to OSI.

After analyzing the cell cycle phase distribution upon treatment with these two inhibitors, it was observed that OSI-906 treatment arrested the cell cycle in the G_1_ phase (Figure 4B), as previously reported by other authors [19]. As a difference, BMS-754807 blocked the cells in the G_2_/M phase and elicited an increase in the fraction of cells in the sub-G_1_ phase (Figure 4A), which corresponded to cell death, an effect that has also been documented in the literature [13]. The fact that both inhibitors exhibit different molecular mechanisms of action points out to the idea that BMS-754807 should bear alternative protein targets accounting for its differential effects as compared to OSI. We have also determined that cell death induced by BMS-754807 likely occurs through caspase-mediated apoptosis, since treatment of cells with a pan-caspase inhibitor in combination with BMS-754807 abrogated cell death induced by the latter (Figure 5). Other studies have shown that BMS-induced cell death occurs through apoptosis mediated by caspase-3 and PARP-1 [13,26]. Considering that we have tried three putative inhibitors of IGF-1R in this article and in our previous article and we have found that two of these inhibitors induced cell death (PPP and BMS) and blocked cell cycle in the G2 + M phase, but the mechanisms of cell death induction are different between them (one is caspase dependent, while the other one is caspase independent), our results suggest that by studying the differences between cell death mechanisms induced by BMS and PPP we have a new putative source to identify new therapeutically approaches for these types of poor diagnosis cancers.

The structure of the catalytic domain of numerous protein kinases is resolved from X-ray diffraction data. It shows the existence of an N-ter and another C-ter lobe that formed a cleft that served as a binding site for ATP and Mg^2+^ [28]. The superfamily of protein kinases shares a catalytic domain with high structural identity [29]. Most kinase inhibitor drugs target this cavity, and this poses a specificity problem when we want to selectively modify the enzymatic activity of a given protein kinase and probably contributes to the development of side effects [28].

To test our hypothesis that BMS-754807 should have alternative targets that are responsible for its greater antiproliferative effect and its differential mechanism of action compared to OSI, we performed cell cycle phase distribution experiments in which we treated cells with an increasing dose of one of the two inhibitors while keeping constant that of the other. If the effect was mediated only by inhibition of IGF-1R and IR, when the concentration of both inhibitors progressively increased, passing from a suboptimal to an optimal concentration capable of completely inhibiting IGF-1R, the same molecular mechanism of action should be seen. However, in both cases, the effect produced by the treatment with BMS-754807 was observed as dominant, so this is a more solid evidence of the presence of alternative targets [30] for BMS-754807 (Figure S3).

In order to study in depth the molecular mechanisms responsible for the effects of BMS-754807, we determined its effect on the activation of several MAP kinases (Figure 6), which are crucial constituent mediators of signaling pathways involved in cell proliferation in response to external stimuli promoting cell growth and stress [30]. The fact that BMS-754807 decreased the activity of ERK1/2 seems reasonable, since MAPKs are involved in the regulation of cell proliferation [30]. This decrease in ERK1/2 phosphorylation could be attributable to its downstream position in the IGF-1R signaling pathway. However, the difference between ERK1/2 inhibition promoted by BMS-754807 (80%) and OSI-906 (40%) was significant, and therefore a distinct mechanism of action should mediate the greater effect of BMS-754807. This inhibitor also increased the phosphorylation of JNK 1 to 3, and in addition decreased the phosphorylation levels of p38α, a critical MAP kinase involved in the immune and inflammatory responses [27]. The effects of this compound on these three types of MAP kinases should overall be responsible, at least in part, for its antiproliferative action and elicited apoptosis.

Results from phosphor-kinase array analysis (Figure 6D) have allowed us to identify several protein kinases differentially regulated by BMS-754807 other than IGF-1R and IR, namely, AMPKα1, mTOR, β-catenin, SRC, FYN, HCK and CHK-2, GSK-3α/β, AKT, PRAS40, AKT1S1 and p38α. In other reports, it has also been stated that BMS-754807 inhibits other protein kinases in addition to IGF-1R and IR [11,13,27], although their identification was not addressed.

AKT and its endogenous ligand PRAS40 regulate metabolism and are involved in cell proliferation and survival [31,32]. Therefore, it seems reasonable that their inhibition should lead to apoptosis [31] as it occurs upon BMS-754807 treatment. In other studies, dephosphorylation of AKT mediated by BMS-754807 [25,26] and by OSI-906 [11,17,19] have been described, although, in our study, OSI inhibited AKT very modestly at the concentrations tested.

GSK-3 is involved in glucose homeostasis, pro-inflammatory effects and cell proliferation [33], and thus its inhibition by BMS is expected to alter cell homeostasis, which should be detrimental to cell survival. Other protein kinases, such as AKT1S1 (a subunit of mTORC1) [32,34], AMPKα1 [35], some kinases of the SRC family [36], FYN [37] and HCK [38] also became inhibited by BMS-754807. Since all these protein kinases are related to processes of cell growth, proliferation and survival, it seems reasonable that upon inhibition of their phosphorylation by BMS or OSI, apoptosis would be induced.

Other proteins differentially inhibited by BMS-754807 were mTOR and CHK-2. mTOR is involved in many cellular processes, including the synthesis of biomolecules and, as the most interesting function for this study, the regulation of cell metabolism, growth and survival [32,39,40]. As for CHK-2, it is a protein kinase known to be related to apoptosis [41,42]. Inhibition of these proteins would thus result in an alteration in cellular homeostasis, so that apoptosis would likely as well be induced.

Finally, we have attempted to unravel the molecular mechanisms that allow BMS, but not OSI, to inhibit their putative target protein kinases. Molecular docking assays were used in this work aimed at predicting the structure of ligand-receptor complexes by means of computational methods and on the basis of high-resolution structural information on the target structure, obtained from X-ray diffraction, NMR neutron scattering spectroscopy, homology modeling and/or molecular dynamics simulations. The software used in this work, Autodock Vina, has a scoring function to make an approximate calculation of the free energy change for interaction between the target (usually a protein) and the ligand in each binding pose [43]. Initially, we performed molecular docking assays for the ATP-binding site of each protein kinase catalytic domain. Values shown in Figure 7 reflect that BMS exhibits a higher affinity than OSI for the ATP-binding site of kinases PTK6, SRC, p38α, mTOR, HCK, GSK-3β and IGF-1R. These data were in agreement with our experimental observations from phospho-RTK arrays showing that while BMS-754807 clearly inhibited these enzymes, OSI-906 did not. By contrast, for other kinases such as AMPK, CHK2, AKT1 and AKT2, the ΔG values for OSI-906 binding were lower than for the BMS-754807, which involves that their affinity for the ATP-binding site would be higher. However, these kinases were not inhibited, or were inhibited to a lesser extent, by OSI than by BMS. This discrepancy could be attributed to different explanations. First, the possibility exists that inhibition of these kinases by BMS is not a direct effect of this compound, but rather a consequence of its promoted inhibition of upstream kinases. Second, an alternative explanation could be that BMS inhibits kinase activity by interacting with an area other than the ATP-binding site. Interestingly, data shown in Figure 8 reflected the presence of BMS binding clusters in the area of intersection between the N- and C-terminal domains of AKT2, AMPK, mTOR, p38α and SRC kinases. It is thus tempting to speculate that the presence of hotspots in this particular area could prevent the opening and closing movement around the catalytic cleft necessary for ATP binding. This would occur in a way similar to that of modulation of JNK1 by JIP1, where the binding of this peptide induces a hinge motion between the N- and C-terminal domains of JNK1 and distorts the ATP-binding cleft, reducing the affinity of this kinase for ATP [44].

Other data proving that the effect of BMS is independent of IGF-1R levels arise from checking the UALCAN database (a comprehensive web resource for analysis of cancer OMICS data) [45]. In this database, when comparing IGF-1R expression levels between tumors and the non-tumor samples from the same tissue, it is observed that, in colon carcinoma, IGF-1R levels are significantly higher in the tumor, whereas, in pancreatic carcinoma, there are no significant differences between tumors and normal tissue, and, in glioblastoma, the expression of IGF-1R is significantly lower in the tumor; yet, we have very significant effects of BMS-754807 on all these models.

BMS-754807 exerts a strong antineoplastic effect on several cancer model cells, apparently in a way mostly independent of IGF-1R inhibition and is able to inhibit the ERK1/2 and PI3K/AKT/mTOR signaling pathways, which are crucial for tumor cells to survive. The identification of the primary targets of BMS responsible for mediating its antiproliferative effects should allow one to develop new compounds able to directly act on them with a potential antineoplastic effect and fewer side effects.

## 4. Materials and Methods

### 4.1. Reagents

The IGF-1R small molecule tyrosine kinase inhibitors BMS-754807 (PubChem 24785538) and OSI-906 (PubChem 11640390) were purchased from Selleck Chemicals (Houston, TX, USA). The pan-caspase inhibitor Z-VAD-FMK was obtained from Calbiochem (La Joya, CA, USA) and used to assess caspase-dependent apoptosis.

### 4.2. Cell Culture

The human colorectal cancer cell lines SW480, SW620, HCT-15 and DLD-1, and pancreatic carcinoma cell lines IMIM-PC-1 (M186), IMIM-PC-2 (M220), HS766T, PANC-1 and RWP-1, were kindly donated by the Instituto Municipal de Investigaciones Médicas (IMIM, Barcelona, Spain). The human glioblastoma cell lines U87-MG (U87), U-87ΔEGFR and T98G (T98) were obtained from the American Type Culture Collection (ATCC, Manassas, VA, USA). HGUE-GB-15, HGUE-GB-16 and HGUE-GB-17 primary GBM cell cultures were obtained from brain aspirates of patients who had been diagnosed with GBM, as previously described [10]. These brain aspirates came from the resection surgery of patients older than 18 years who had signed the corresponding informed consent. The procedures for obtaining tissue samples were developed in accordance with national ethical and legal standards, and following the guidelines established in the Declaration of Helsinki (2000). The present research project was conducted under the written approval of the Clinical Research Ethics Committee of the Hospital General Universitario de Elche (Spain) and in collaboration with the hospital’s biobank, which is integrated in the Valencian Biobanks Network. HGUE-GB-16 was finally developed into an established human GBM cell line [10].

All cell lines were maintained in Dulbecco’s modified Eagle’s medium (DMEM) High Glucose (Biowest, Riverside, MO, USA) supplemented with 10% (*v/v*) heat-inactivated fetal bovine serum (FBS) and 1% (*v/v*) of a penicillin and streptomycin mixture (also from Biowest) and incubated at 37 °C in a humidified 5% (*v/v*) CO_2_ atmosphere. Primary cultures were maintained under the same conditions previously described, except that the medium was supplemented with F-12 Nutrient Mixture (Gibco, Life technologies, Carlsbad, CA, USA).

### 4.3. Cell Proliferation Assays

Cells were seeded in 96-well plates (Sarstedt, Nümbrecht, Germany) at a density of 4000 cells per well and incubated at 37 °C with high humidity and 5% CO_2_ for 24 h. Then, the indicated compounds were added in sextuplicate, and each plate was incubated under culture conditions for 72 h. Thereafter, methylthiazolyldiphenyl-tetrazolium bromide (MTT) (Sigma-Aldrich, St. Louis, MO, USA) was added at 0.25 mg^−1^, and after incubation for 3 h the medium was removed and 200 μL of dimethyl sulfoxide (DMSO) (Sigma-Aldrich) was added. After shaking at room temperature for 30 min to dissolve the formazan crystals [46], the absorbance at 570 nm was measured on a BioTek Gen5™ (Winooski, VT, USA) microplate reader.

### 4.4. Cell Cycle Phase Distribution

Cells were seeded in 6-well plates (Sarstedt, Nümbrecht, Germany), and after treatments they were harvested by trypsinization and fixed in cold ethanol (75%; *v/v*) for at least 30 min at −20 °C. Thereafter, the cells were pelleted, resuspended in 0.5 mL of phosphate-buffered saline (PBS) in the presence of 0.5% Triton X-100, 25 µg mL^−1^ RNase A (Serva, Heidelberg, Germany) and 25 µg mL^−1^ propidium iodide (Sigma Aldrich), and incubated for 30 min at room temperature in the dark. Finally, the cell cycle phase distribution was determined as a function of the cellular DNA content in a BD FACSCanto™ flow cytometer (Becton Dickinson, Franklin Lakes, NJ, USA).

### 4.5. MAPK Phosphorylation

The RWP-1 pancreatic adenocarcinoma cell line was treated with BMS-754807 or OSI-906 for 1 or 6 h. Then, the culture medium was discarded, cells were washed with PBS and cell extracts were obtained to be assayed for phosphorylated MAPK levels using the InstantOne ELISA kit from eBioscience (San Diego, CA, USA), following the manufacturer’s instructions. Briefly, the cells were lysed in an appropriate volume of the cell lysis mix provided in the kit (100 µL for a 96-well plate) with shaking (300 rpm) at room temperature for 10 min. Then, 50 µL of cell lysate was added to each well of InstantOne ELISA microplates and, after the addition of 50 µL of the antibody cocktail, incubation was carried out for 1 h. Fifty µL of cell lysis mix or of the control lysate provided in the kit were used as negative and positive controls, respectively. Thereafter, three washes with 200 µL of wash buffer per well each time were carried out, and 100 µL of detection reagent was finally added. After shaking for 30 min, stop solution was added and the absorbance was read at 450 and 650 nm.

### 4.6. Phospho-RTK Array Analysis

RWP-1 cells were treated with BMS-754807 or OSI-906 for 6 h, and then harvested and analyzed for levels of phosphorylated tyrosine kinases using the Human Phospho-RTK Array Kit (Figure 1) or Human Phospho-Kinase Array Kit (Figure 6), both from R&D Systems (Minneapolis, MN, USA) following the manufacturer’s instructions. Briefly, the array membranes were blocked with 1 mL of array buffer, and then 1 mL of cell lysate was added to each blot and incubation was carried out overnight at 2–8 °C on a rocking platform. Next, the blots were washed three times with 20 mL of wash buffer, 1 mL of the detection anti-phosphotyrosine antibody cocktail was added, and the blots were incubated for 2 h at room temperature. Following a new round of washes, 1 mL of diluted streptavidin-HRP solution was added and incubated for 30 min at room temperature. Finally, after a new wash round, 1 mL of chemical reagent mix was added to the membranes, and images were obtained after X-ray film exposure.

### 4.7. Molecular Docking Simulations

To date, a number of crystal structures of the catalytic domain of protein kinases analyzed in this study have been solved and deposited in the Protein Data Bank (PDB). A complete list of all structures used in assays of molecular docking against the catalytic site of these protein kinases is included in Table S1. For each model, H_2_O molecules, ions and inhibitors were removed. The addition and visualization of protein structures and the elaboration of figures were carried out using the PyMOL v.2.3 software from the Schrödinger LLC platform (PyMOL Molecular Graphics System, v2.3.3 Schrödinger, New York, NY, USA, LLC, at http://www.pymol.org/).

Molecular docking assays of ligands (BMS and OSI) against the ATP-binding site of each protein kinase catalytic domain were carried out as previously described [23,24,47]. Briefly, all the selected protein structures were subjected to geometry optimization using the repair function of the FoldX protein design algorithm [48,49]. To perform molecular docking with the AutoDock/vina, the receptor and ligand structures were formatted as PDBQT files. A grid with dimensions of 23 × 23 × 23 points was centered to ensure coverage of the binding site of the structure. AutoDock/vina was set up on a Linux cluster at the Cluster of Scientific Computing (http://ccc.umh.es/) of the Miguel Hernández University (UMH). The chemical structures of BMS-754807 and OSI-906 were retrieved from the NCBI PubChem Compound database [50]. To search for potential binding sites for these two drugs other than the ATP-binding domain, a global molecular docking procedure was performed with AutoDock/vina implemented in YASARA Structure v19.9.17 (Vienna, Austria) [51], where a total of 500 flexible docking runs were set and clustered around the putative binding sites. With this purpose, the AMBER-99 forcefield was used [52].

### 4.8. Statistic Analysis

The results are shown as the mean ± standard error of the mean (SEM) of at least three independent experiments. A descriptive statistic was performed with the GraphPad Prism version 5 (GraphPad Software Inc., San Diego, CA, USA) calculating the mean, standard error of the mean, and standard deviation for the values. The Shapiro–Wilk statistical test was used to evaluate the normal distribution of the data, and, to analyze the association between variables, Student’s *t*-test or the Mann–Whitney U test was used. *p*-values lower than 0.05 were considered statistically significant.

## 5. Conclusions

Although OSI and BMS are able to inhibit IGF-1R activity at low doses, the differential effect shown by both compounds against colon, pancreatic and glioblastoma cancer cellular models is mediated by BMS off-target effects. Since BMS has an important antineoplastic effect on these poor prognosis types of cancer, this compound could be taken into consideration for treatment independently of IGF-1R status.

We have identified several kinases that can mediate the BMS off-target effects. Interestingly, our data suggest that BMS could affect these kinases not only blocking their ATP-binding domain, but also by means of allosteric interactions.

The differential molecular mechanisms of cell death induction evoked by BMS and PPP (another IGF-1R inhibitor) on the same cellular models open the possibility to find a new therapeutic target for poor prognosis cancers based on these molecular mechanisms of cell death.

## Supplementary Materials

The following are available online at https://www.mdpi.com/2072-6694/12/12/3717/s1, Table S1: List of PDB IDs of the structures analyzed for ligand binding by molecular docking assays carried out for different protein kinases. The ΔG values plotted in Figure 7 result from averaging the values obtained in molecular docking simulations for all the structures listed in the table for each protein analyzed. For each PDB entry, the experimental method (X-ray diffraction data only), the resolution (in Ǻ), and the position of amino acids in the resolved structure are indicated, Figure S1: Effect of BMS-754807 and OSI-906 on IGF-1R phosphorylation in RWP-1. Cell extracts non-treated (control), or pre-treated with 500 nM (panel A) or 10 µM (panel B) BMS-754807 or OSI-906 for 6 h were subjected to Western blot analysis using antibodies against phospho-IGF-1R (Tyr 1161 from signal way antibody# 11087) and IGF-1R (Santa Cruz Biotechnology sc-713), Figure S2: Dose–response effect of BMS-754807 and OSI-906 on cell proliferation in RWP-1 cell line. The indicated cell line was treated with 0.01–10 µM BMS-754807 or OSI-906 for 72 h and cell proliferation was evaluated by the MTT assay, Figure S3: Effect of BMS-754807 and OSI-906 on cell cycle phase distribution in the IMIM-PC-2 pancreatic carcinoma cell line. Cells were treated with increasing concentrations (1 to 10 μM) of BMS-754807 or OSI-906, in the presence of a constant concentration (1 μM) of the alternative inhibitor (OSI-906 or BMS-754807 respectively) for 24 h. Then, the percentage of cells in each phase of the cell cycle was analyzed by flow cytometry. Data represent the mean ± SEM (*n* ≥ 3) of the percentage of treated minus that of untreated cells.
