# Supplementary material for: Differential Effects of IGF-1R Small Molecule Tyrosine Kinase Inhibitors BMS-754807 and OSI-906 on Human Cancer Cell Lines
Source: Cancers (Basel). 2020 Dec 11;12(12):3717. doi: 10.3390/cancers12123717 (PMC7763458; doi:10.3390/cancers12123717)
Supplement: Supplementary file 1 [file cancers-12-03717-s001.pdf]

Supplementary Materials: Differential Effects of IGF-1R Small Molecule Tyrosine Kinase Inhibitors BMS-754807 and OSI-906 on Human Cancer Cell Lines

María Fuentes-Baile, María P. Ventero, José A. Encinar, Pilar García-Morales, María Poveda-Deltell, Elizabeth Pérez-Valenciano, Víctor M. Barberá, Javier Gallego-Plazas, Álvaro Rodríguez-Lescure, José Martín-Nieto and Miguel Saceda

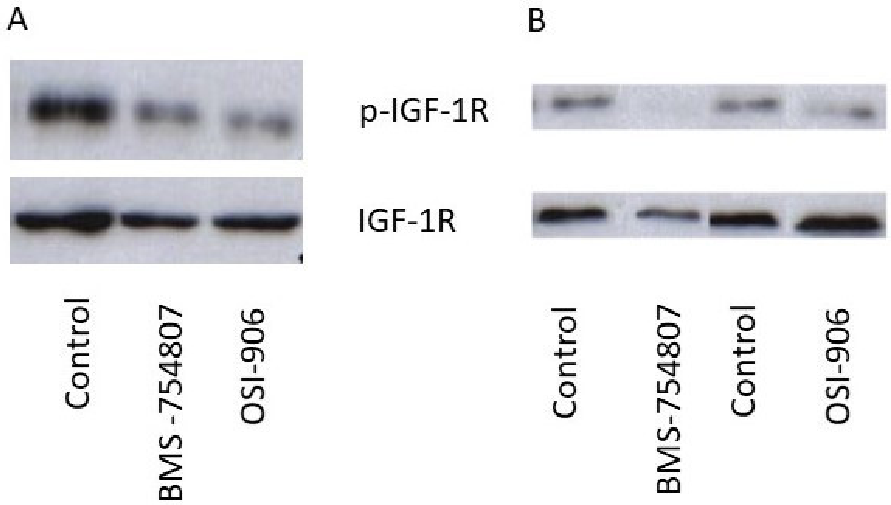

Figure S1. RWP-1 cell extracts cells were grown in 10% FBS-containing media (FBS). Then, cells were non-treated (control), or pre-treated with 500 nM (**panel A**) or 10 µM (**panel B**) BMS-754807 or OSI-906 for 6 hours. Proteins were extracted and subjected to Western blot using antibodies against phospho-IGF-1R (Tyr 1161 from Signalway Antibody# 11087) and IGF-1R (Santa Cruz Biotechnology sc-713).

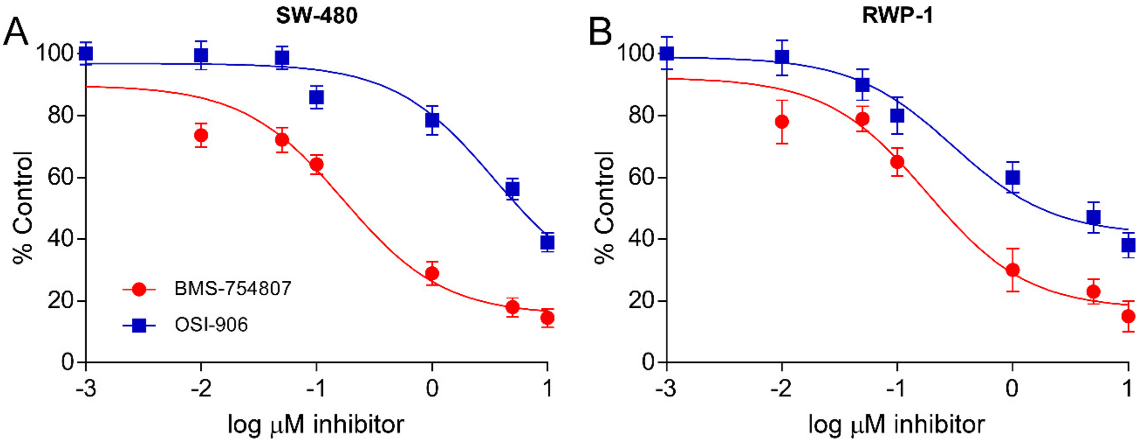

Figure S2. Dose-response effect of BMS-754807 and OSI-906 on cell proliferation in SW480 and RWP-1 cell lines. The indicated cell lines were treated with 0.01-10 µM BMS-754807 or OSI-906 for 72 h and cell proliferation was evaluated by the MTT assay. Data represent the mean ± SEM ($n \geq 6$) of viable cells percentage with respect to untreated controls, taken as 100%.

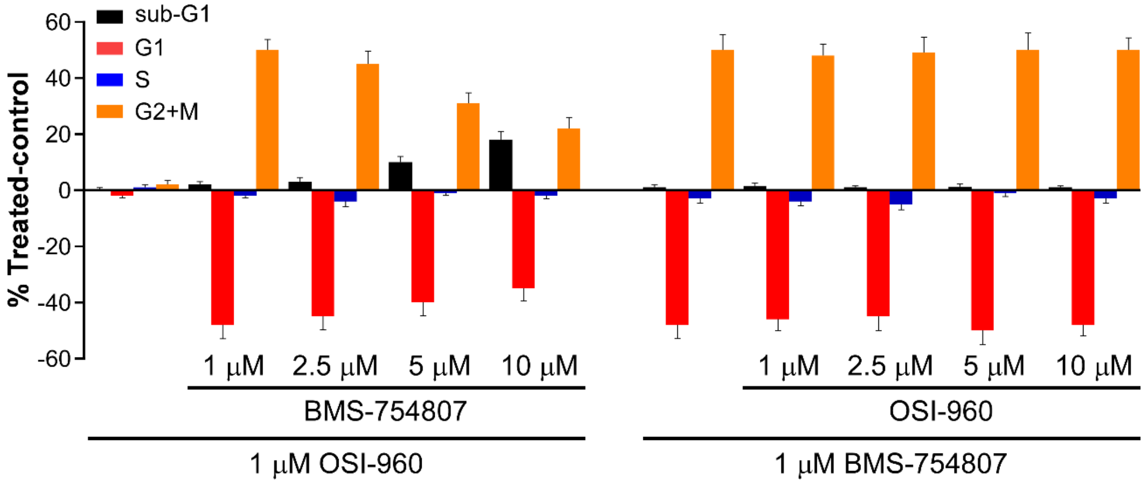

Figure S3. BMS-754807 and OSI-906 effect on cell cycle distribution on IMIM-PC-2 pancreatic carcinoma cell line. The IMIM-PC-2 cells were treated with increasing concentrations 1–10 μM of OSI-906 or BMS-754807, in the presence of a constant concentration of 1 μM of the alternative inhibitor (BMS-754807 or OSI-906 respectively) for 24 h and cell cycle distribution was analyzed by flow cytometry. Data represent the mean ± SEM of the percentages of treated cells minus those of the control cells with a $n \geq 3$.

Table S1. List of PDB codes of the structures used in the molecular docking experiments for the different protein kinases analyzed in this study. The results of ΔG included in Figure 7 arise from averaging the values obtained in molecular docking experiments with all the structures listed in the table for each protein analyzed. For each PDB entry, the experimental method (X-ray diffraction data only), the resolution A, and the position of the amino acids of the resolved structure are included.

**Protein:** PTK6, **UniProt code:** Q13882			
PDB entry	Method	Resolution (Å)	Positions
5D7V	X-ray	2.33	185–446
5DA3	X-ray	1.7	185–446
5H2U	X-ray	2.24	185–446

**Protein**: mTor, **UniProt code**: P42345			
PDB entry	Method	Resolution (Å)	Positions
4JSN	X-ray	3.2	1376–2549
4JSP	X-ray	3.3	1376–2549
4JSV	X-ray	3.5	1376–2549
4JSX	X-ray	3.5	1376–2549
4JT5	X-ray	3.45	1376–2549
4JT6	X-ray	3.6	1376–2549
5WBU	X-ray	3.42	1376–2549
5WBY	X-ray	3.1	1376–2549

**Protein**: CHK2, **UniProt code**: O96017			
PDB entry	Method	Resolution (Å)	Positions
2CN5	X-ray	2.25	210–531
2CN8	X-ray	2.7	210–531
2W0J	X-ray	2.05	210–531
2W7X	X-ray	2.07	210–531
2WTC	X-ray	3	210–531
2WTD	X-ray	2.75	210–531
2WTI	X-ray	2.5	210–531
2WTJ	X-ray	2.1	210–531

**Protein:** SRC, **UniProt code:** P12931			
PDB entry	Method	Resolution (Å)	Positions
2H8H	X-ray	2.2	2–536
1YOJ	X-ray	1.95	254–536
1YOL	X-ray	2.3	254–536
1YOM	X-ray	2.9	254–536
4MXO	X-ray	2.1	254-536
4MXX	X-ray	2.6	254–536
4MXY	X-ray	2.58	254–536
4MXZ	X-ray	2.58	254–536
2BDF	X-ray	2.1	258–536
2BDJ	X-ray	2.5	258–536
1YI6	X-ray	2	261–536
1FMK	X-ray	1.5	86–536
1KSW	X-ray	2.8	86–536
1Y57	X-ray	1.91	86–536
2SRC	X-ray	1.5	86–536
4K11	X-ray	2.3	87-534

**Protein**: HCK, **UniProt code**: P08631			
PDB entry	Method	Resolution (Å)	Positions
5H0B	X-ray	1.65	81–526
5H0H	X-ray	1.72	81–526
5H0G	X-ray	1.8	81–526
5H09	X-ray	1.95	81–526
2HK5	X-ray	2	247–514
1QCF	X-ray	2	81–526

2XBJ	X-ray	2.3	210–531
2XK9	X-ray	2.35	210–531
2XM8	X-ray	3.4	210–531
2XM9	X-ray	2.5	210–531
2YCF	X-ray	1.77	210–530
2YCQ	X-ray	2.05	210–531
2YCR	X-ray	2.2	210–531
2YCS	X-ray	2.35	210–531
2YIQ	X-ray	1.89	210–531
2YIR	X-ray	2.1	210–531
2YIT	X-ray	2.2	210–531
3I6U	X-ray	3	84–502
3I6W	X-ray	3.25	70–512
4A9R	X-ray	2.85	210–531
4A9S	X-ray	2.66	210–531
4A9T	X-ray	2.7	210–531
4A9U	X-ray	2.48	210–531
4BDA	X-ray	2.6	210–531
4BDB	X-ray	2.5	210–531
4BDC	X-ray	3	210–531
4BDD	X-ray	2.67	210–531
4BDE	X-ray	2.55	210–531
4BDF	X-ray	2.7	210–531
4BDG	X-ray	2.84	210–531
4BDH	X-ray	2.7	210–531
4BDI	X-ray	2.32	210–531
4BDJ	X-ray	3.01	210–531
4BDK	X-ray	3.3	210–531

Protein: AKT2, **UniProt code**: P31751

PDB entry	Method	Resolution (Å)	Positions
1GZK	X-ray	2.3	146–460
1GZN	X-ray	2.5	146–480
1GZO	X-ray	2.75	146–460
1MRV	X-ray	2.8	143–481
1MRY	X-ray	2.8	143–481
1O6K	X-ray	1.7	146–481
1O6L	X-ray	1.6	146–467
2JDO	X-ray	1.8	146–467
2JDR	X-ray	2.3	146–467
2UW9	X-ray	2.1	146–467
2X39	X-ray	1.93	146–467
2XH5	X-ray	2.72	146–479
3D0E	X-ray	2	146–480
3E87	X-ray	2.3	146–480
3E88	X-ray	2.5	146–480
3E8D	X-ray	2.7	146–480

Protein: P38 alpha, **UniProt code**: Q16539

PDB entry	Method	Resolution (Å)	Positions
2FST	X-ray	1.45	2–360
3LFF	X-ray	1.5	2–360
3OEF	X-ray	1.6	1–360
5WJJ	X-ray	1.6	1–360
3ZS5	X-ray	1.6	2–360
4EHV	X-ray	1.6	2–360

5H0E	X-ray	2.1	81–526
2C0T	X-ray	2.15	81–526
3VS3	X-ray	2.17	81–526
3VRZ	X-ray	2.22	81–526
2C0I	X-ray	2.3	81–526
3VS6	X-ray	2.37	81–526
3VS1	X-ray	2.46	81–526
3VRY	X-ray	2.48	81–526
1AD5	X-ray	2.6	79–526
3VS2	X-ray	2.61	81–526
3VS4	X-ray	2.75	81–526
2C0O	X-ray	2.85	81–526
3VS5	X-ray	2.85	81–526
4LUD	X-ray	2.85	81–526
3VS0	X-ray	2.93	81–526
2HCK	X-ray	3	79–526
3VS7	X-ray	3	81–526
4LUE	X-ray	3.04	81–526

Protein: FYN, **UniProt code**: P06241

PDB entry	Method	Resolution (Å)	Positions
2DQ7	X-ray	2.8	261–537

Protein: AKT1, **UniProt code**: P31749

PDB entry	Method	Resolution (Å)	Positions
6CCY	X-ray	2.18	144–466
3CQU	X-ray	2.2	144–480
3CQW	X-ray	2	144–480
3MV5	X-ray	2.47	144–480
3MVH	X-ray	2.01	144-480
3OCB	X-ray	2.7	144–480
3OW4	X-ray	2.6	144–480
3QKK	X-ray	2.3	144–480
3QKL	X-ray	1.9	144–480
3QKM	X-ray	2.2	144–480
4EKK	X-ray	2.8	144–480
4EKL	X-ray	2	144–480
4GV1	X-ray	1.49	144–480
3O96	X-ray	2.7	2–443
4EJN	X-ray	2.19	2–446
5KCV	X-ray	2.7	2–446

Protein: GSK-3 beta, **UniProt code**: P49841

PDB entry	Method	Resolution (Å)	Positions
1J1B	X-ray	1.8	1–420
1Q5K	X-ray	1.94	7–420
4AFJ	X-ray	1.98	27–393
4PTE	X-ray	2.03	1–420
4NM3	X-ray	2.1	1-383
1J1C	X-ray	2.1	1–420
1Q41	X-ray	2.1	2–420
3DU8	X-ray	2.2	1–420
1Q3D	X-ray	2.2	2–420
5K5N	X-ray	2.2	28–384
4ACC	X-ray	2.21	1–420
3SAY	X-ray	2.23	1–420

4GEO	X-ray	1.66	2–360
3FMK	X-ray	1.7	1–360
3ROC	X-ray	1.7	1–360
5XYY	X-ray	1.7	1–360
2FSL	X-ray	1.7	2–360
2QD9	X-ray	1.7	2–360
3K3I	X-ray	1.7	5–352
2RG6	X-ray	1.72	2–360
2GFS	X-ray	1.75	2–360
2ZAZ	X-ray	1.8	1–360
3FL4	X-ray	1.8	1–360
3FLY	X-ray	1.8	1–360
3GC7	X-ray	1.8	1–360
3KQ7	X-ray	1.8	1–360
5MTX	X-ray	1.8	1–360
1WBS	X-ray	1.8	2–360
2NPQ	X-ray	1.8	2–360
3HUC	X-ray	1.8	2–360
4AA0	X-ray	1.8	2–360
3S3I	X-ray	1.8	4–352
2FSO	X-ray	1.83	2–360
5N68	X-ray	1.85	1–360
4F9Y	X-ray	1.85	2–360
2FSM	X-ray	1.86	2–360
4E5A	X-ray	1.87	1–360
3HL7	X-ray	1.88	1–360
3NNW	X-ray	1.89	1–354
3MPT	X-ray	1.89	1–360
3ZSG	X-ray	1.89	2–360
3D83	X-ray	1.9	1–360
3FLN	X-ray	1.9	1–360
3FLQ	X-ray	1.9	1–360
3FMH	X-ray	1.9	1–360
3FMN	X-ray	1.9	1–360
3ZYA	X-ray	1.9	1–360
5ML5	X-ray	1.9	1–360
5N67	X-ray	1.9	1–360
4DLI	X-ray	1.91	2–360
2Y8O	X-ray	1.95	1–360
3HLL	X-ray	1.95	1–360
3HV6	X-ray	1.95	2–360
3CTQ	X-ray	1.95	5–352
4KIN	X-ray	1.97	2–360
1R3C	X-ray	2	1–360
1ZYJ	X-ray	2	1–360
1ZZ2	X-ray	2	1–360
2I0H	X-ray	2	1–360
3E92	X-ray	2	1–360

1R0E	X-ray	2.25	35–420
4NM5	X-ray	2.3	13–383
4NM7	X-ray	2.3	13–383
1Q3W	X-ray	2.3	2–420
3I4B	X-ray	2.3	7–420
4J71	X-ray	2.31	1–420
2JLD	X-ray	2.35	1–420
4PTG	X-ray	2.36	1–420
3ZRK	X-ray	2.37	23–393
1PYX	X-ray	2.4	1–420
5KPK	X-ray	2.4	1–420
3GB2	X-ray	2.4	34–383
3F7Z	X-ray	2.4	35–383
1O9U	X-ray	2.4	35–384
5HLP	X-ray	2.45	1–420
3ZRL	X-ray	2.48	23–393
3ZRM	X-ray	2.49	23–393
4NM0	X-ray	2.5	1–383
5F94	X-ray	2.51	36–385
5F95	X-ray	2.52	36–385
4ACD	X-ray	2.6	1–420
4ACG	X-ray	2.6	1–420
4ACH	X-ray	2.6	1–420
5KPL	X-ray	2.6	1–420
6B8J	X-ray	2.6	1–420
1GNG	X-ray	2.6	27–393
4DIT	X-ray	2.6	27–393
3F88	X-ray	2.6	35–383
3ZDI	X-ray	2.64	35–384
5KPM	X-ray	2.69	1–420
1I09	X-ray	2.7	1–420
4J1R	X-ray	2.7	1–420
3Q3B	X-ray	2.7	2–420
3SD0	X-ray	2.7	35–384
4PTC	X-ray	2.71	1–420
1Q4L	X-ray	2.77	2–420
4B7T	X-ray	2.77	35–384
1UV5	X-ray	2.8	35–384
1H8F	X-ray	2.8	35–386
2OW3	X-ray	2.8	35–386
5T31	X-ray	2.85	1–420
3L1S	X-ray	2.9	7–420
3PUP	X-ray	2.99	1–420
5HLN	X-ray	3.1	1–420
4IQ6	X-ray	3.12	1–420
3M1S	X-ray	3.13	1–420
5OY4	X-ray	3.2	1–420
2O5K	X-ray	3.2	29–393

Protein: IGF-1R, **UniProt code**: P08069

PDB entry	Method	Resolution (Å)	Positions
1P4O	X-ray	1.5	974–1294
3LW0	X-ray	1.79	983–1286
5FXS	X-ray	1.9	980–1286
2OJ9	X-ray	2	982–1286
3I81	X-ray	2.08	982–1286

Protein: IGF-1R, **UniProt code**: P08069

PDB entry	Method	Resolution (Å)	Positions
4CFE	X-ray	3.02	1–552
4CFF	X-ray	3.92	1–552
4ZHX	X-ray	2.99	2–552
5ISO	X-ray	2.63	1–552
6B1U	X-ray	2.77	2–552

1JQH	X-ray	2.1	979–1286	6B2E	X-ray	3.8	2–552
3O23	X-ray	2.1	982–1286				
4D2R	X-ray	2.1	985–1286				
1K3A	X-ray	2.1	988–1286				
3NW7	X-ray	2.11	982–1286				
3NW5	X-ray	2.14	982–1286				
3NW6	X-ray	2.2	982–1286				
5HZN	X-ray	2.2	983–1286				
5FXQ	X-ray	2.3	980–1286				
3D94	X-ray	2.3	986–1286				
5FXR	X-ray	2.4	980–1286				
2ZM3	X-ray	2.5	981–1286				
1M7N	X-ray	2.7	974–1294				
3F5P	X-ray	2.9	981–1286				
3QQU	X-ray	2.9	988–1286				
3LVP	X-ray	3	951–1286				

Publisher's Note: MDPI stays neutral with regard to jurisdictional claims in published maps and institutional affiliations.

© 2020 by the authors. Licensee MDPI, Basel, Switzerland. This article is an open access article distributed under the terms and conditions of the Creative Commons Attribution (CC BY) license (http://creativecommons.org/licenses/by/4.0/).
